# Prime editing: Emerging mechanisms, engineering innovations, and next-generation applications

**DOI:** 10.1016/j.bidere.2026.100073

**Published:** 2026-03-20

**Authors:** Waqar Muhammad, Ali Amjad, Yufei Liu, Aziz Umar, Kai Chen, Xiaolong Wang, Kun Xu

**Affiliations:** a Hainan Institute of Northwest A&F University, Sanya, 572025, Hainan, China; b College of Animal Science and Technology, Northwest A&F University, No.22 Xinong Road, Yangling, 712100, Shaanxi, China; c Department of Poultry Production, Government of Sindh, Pakistan; d College of Life Sciences, Northwest A&F University, No.22 Xinong Road, Yangling, 712100, Shaanxi, China

**Keywords:** Prime editing, pegRNA, Genome rewriting, Mismatch repair, Gene correction, Recombinase integration, Precision genome engineering

## Abstract

Prime editing has become a highly programmable and accurate genome-editing platform that can install targeted substitutions, insertions, and deletions without introducing double-strand breaks or requiring a separate donor DNA template. This review summarizes recent developments about prime editing mechanisms, such as knowledge about flap dynamics, repair pathway interactions, and pegRNA architecture, and improvements in engineering, resulting in high-efficiency systems, including PEmax, PE4/5, TWIN-PE, PASTE, and PrimeRoot. Such advances now make prime editing applicable to therapeutic gene correction, agricultural biotechnology, microbial engineering, and functional genomics. However, delivery, chromatin context, mismatch-repair variability, and large-fragment integration remain major barriers to broad application. By comparing prime editing with other genome-editing modalities, this review summarizes its unique advantages and highlights strategic innovations needed for its next stage of development. Together, these developments position prime editing as a highly programmable platform with strong potential to shape the future of precise genome rewriting.

## 1 Introduction

Recent progress in genome engineering has been largely driven by the development of CRISPR-based technologies, which have significantly improved the ability to modify genetic sequences with precision. The original CRISPR/Cas9 system enabled targeted genome editing through the introduction of double-stranded DNA breaks [[1], [2], [3]]. Later improvements led to the development of Cas9 nickase variants [4], and base editing systems such as cytosine base editors (CBE) [5], and adenine base editors (ABE), which allow precise nucleotide substitutions without generating double-strand breaks [6]. Further advancements expanded the genome editing toolbox with prime editing systems (PE1-PE3) [7], and their optimized variants, including PE4-PE5 and PEmax [8], as well as PAM-expanded editors (SpG/SpRY) [9,10], Twin Prime Editing (TwinPE) [11], and the PASTE system for more complex DNA insertions [12].

Prime editing has quickly become one of the most practical, accurate, and programmable genome-editing technologies developed to date. Compared to the traditional CRISPR-Cas systems, which utilise either double-stranded DNA breaks or single-base deamination chemistries, prime editing offers the advantages of installing targeted replacements, small-scale insertions, specific deletions, and gene repair without the need for a donor DNA template or double-stranded cleavage. This type of nickase version of Cas9 is coupled with a reverse transcriptase and a specially designed prime editing guide RNA (pegRNA), a novel form of rewriting genetic information [[13], [14], [15]]. Over the past few years, prime editing has undergone multiple stages of engineering development, starting as an original conceptual system and evolving into ever-stronger models such as PE2, PE3, PE4, PE5, PEmax, TwinPE, PASTE, and PrimeRoot. Protein engineering, refinements of RNA structures, modulation of DNA repair, and protein integration can be combined to launch a new wave of genome-writing platforms, as demonstrated by this platform's transformation [16,17].

This review focuses on mechanistic insights, engineering innovations, and emerging applications of prime editing in biology, medicine, agriculture, and microbial systems. We emphasise how successive design iterations have converted prime editing from a conceptual tool into a programmable genome-rewriting platform. When numerous reviews have characterised prime editing as a technical development, here it is reframed as a programmable genome-rewriting platform capable of multi-step sequence modification. Also discusses both the evolution of prime-editing mechanisms and the emerging frontiers of their biological applications. The idea is to introduce one coherent and novel entire point of view, Prime Editing, which showcases what the existing system does, and how it can be designed to be implemented in the new generation of high-precision and efficient genome editing [14].

The significance of prime editing goes far beyond its technical abilities in the present moment. It opens the possibility that it is possible to rewrite the DNA with an internal RNA template, that cellular repair outcomes can be guided by endogenous repair programs rather than driven by double-strand breaks and that alterations to the genes can be delivered with never-before-promised predictability. This conceptual shift elevates prime editing beyond a CRISPR alternative, positioning it as a modular and programmable paradigm for precision genome engineering. As agricultural technology and microbial engineering change to translational therapeutic application, it is important that a clear understanding of both its mechanistic basis and engineering parameters is essential [7,8].

## 2 Mechanistic foundations of prime editing

**Fig. 1:**
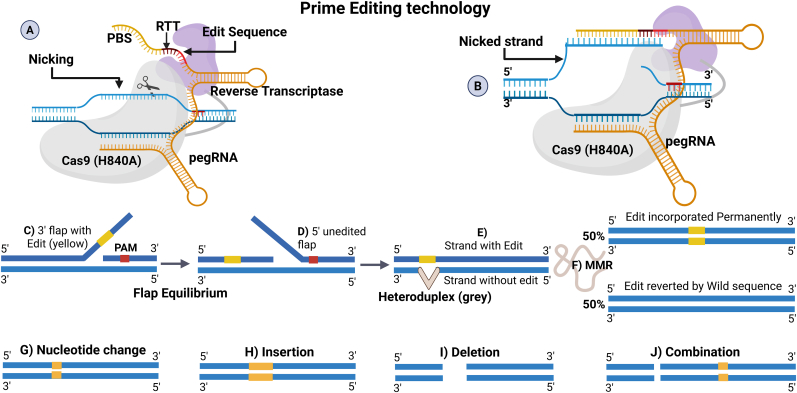
**Mechanism of Prime Editing.** Stepwise process showing Cas9 nicking, primer binding site hybridization, reverse transcription of the edit sequence, 3′ and 5′ flap equilibration, heteroduplex formation, mismatch repair dynamics, and final editing outcomes including substitution, insertion, and deletion.

Prime editing proceeds through a series of steps DNA targeting, nicking, reverse transcription, flap equilibration, and interaction with DNA-repair pathways. The basic molecular reactions in the centre of the system are shown in Fig. 1. Its prime editor consists of a Cas9 nick containing a reverse transcriptase, which is modified to form a nick, followed by a pegRNA with a primer-binding site and a reverse transcription template that encodes the desired edit. When the nick is precisely positioned at the target site by the Cas9 nick, the pegRNA binds its primer binding sequence to the released DNA, after which the reverse transcriptase synthesises a DNA strand containing the intended edit. The outcome of this process is the creation of two competing flaps, namely a 3′ flap with the new edited sequence and a 5′ flap with the old genomic sequence. The cellular repair machinery must then choose whether to incorporate the newly synthesised 3′ flap or retain the original 5′ flap sequence [7,8,18,19]. The rate of editing, therefore, is highly dependent on the thermodynamic and kinetic features of flap stability and the dynamics of DNA repair mechanisms. Flap ‘favoritisms’ likely reflects differences in flap stability (length and base composition), mismatch positioning within the heteroduplex, and competition between flap processing/ligation and mismatch repair. Consequently, edits that stabilize the 3′ flap or reduce effective mismatch recognition are more likely to be retained, while strong mismatch recognition can bias outcomes toward reversion to the original 5′ sequence [7,20].

**Fig. 2:**
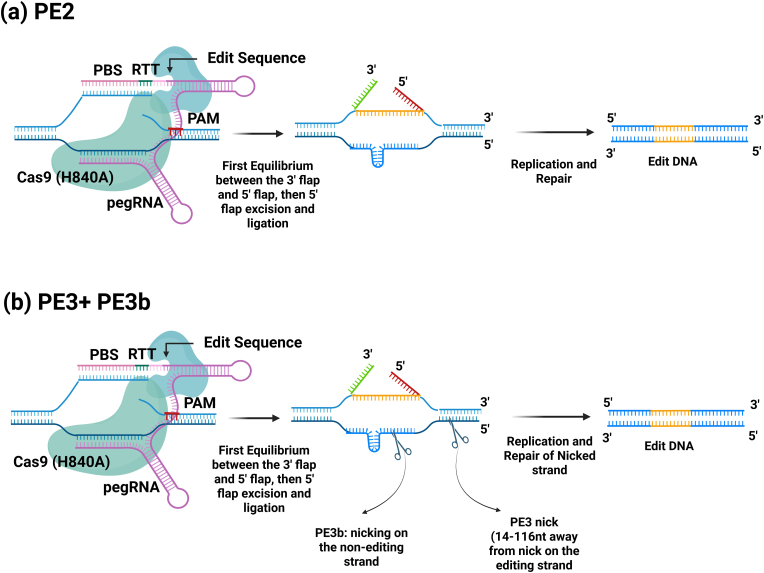
**Mechanisms of PE2 and PE3/PE3b prime editing. (a)** PE2 generates a 3′ flap containing the intended edit, which competes with the endogenous 5′ flap during repair. **(b)** PE3/PE3b introduces a second nick on the non-edited strand, biasing repair toward the edited flap and increasing editing efficiency.

Recent work shows that prime editing is not a simple deterministic reaction, but a dynamic interplay between flap structures and DNA repair pathway processes. Depending on the resolution of the heteroduplex, the mismatch repair system either promotes or suppresses the intended edit. This competition between mismatch repair and flap resolution is the main reason why the orientation of the nick strongly affects editing outcomes [8,20]. Fig. 1 indicates that the edited template and the wild-type template are present in a heteroduplex state, and the mismatch repair system may either retain or eliminate the intended modification. Prime editing systems were more advanced than this mechanistic base. Fig. 2 shows how the original PE2 system was refined into the PE3 system. PE2 had presented a reverse transcriptase engineered that has better catalytic characteristics and makes the initial step of DNA synthesis more efficient [7,11,21]. Nevertheless, editing was restricted because it competed with the wild, unrepaired type of strand. The PE3 system solved this by creating a second nick in the non-edited strand, which favoured repairing the strand with the edited sequence rather than the wild-type one. Mechanistically, PE3 uses an additional nicking guide RNA to nick the non-edited strand, biasing repair/replication toward copying the edited strand. PE3b schedules the nick to occur preferentially after editing, which can reduce indels while still enriching for the desired edit [7,8,12,14]. This little addition greatly enhanced the effectiveness of editing in a large number of targets. However, with the second nick added, a slight possibility of indels has come up once again, and this explains the significance of the balance between maximizing editing efficiency and minimizing nick-induced indels [22,23].

**Fig. 3:**
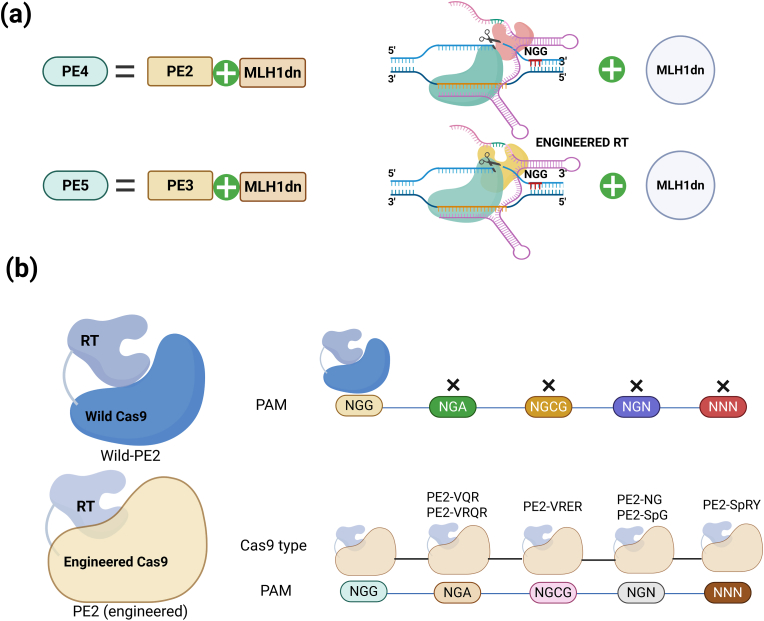
**Engineering enhancements in PE4/PE5 and PAM-flexible PE2 variants. (a)** PE4 and PE5 incorporate MLH1dn to suppress mismatch repair and boost editing efficiency. **(b)** Engineered Cas9 variants expand PAM compatibility, enabling broader prime-editing target ranges.

At an additional level of refinements that are not mechanistic in nature, one more important area of enhancement is the type of Cas9 variants that have an expansion or altered PAM recognition. Prime editing with the original SpCas9(H840A) is limited to NGG PAM -locations. additional variations that allow NGA, NGCG, NGN or complete flexible PAM recognition greatly broaden the available genetic landscape [9,24]. as shown in Fig. 3b. This expansion is conceptualized with an equation-like framework in terms of effective coverage in the genome, and it grows non-linearly with the relaxation of PAM constraints. The most recent designs of prime editing are therefore more about incorporating broad-PAM Cas9 variants, where the targeting can be set by the user with few genomic limitations. The mechanistic principles of prime editing show the existence of a fine balance between RNA-instructed DNA nickase, template-controlled DNA replication, and cell DNA repair signatures [[25], [26], [27]]. These interactions can be best comprehended since they affect the design of a functional prime editor, the result, and the decrease of unwanted by-products. The engineering innovations that are discussed in the next section are also grounded in this [28,29].

## 3 Engineering innovations and advanced prime editing systems

The combination of protein engineering, DNA-repair modulation, guide-RNA structural design, and codon optimization has advanced prime editing into more efficient and versatile systems [30]. The development of structural and functional innovations leading to PE4, PE5, PEmax, and their variations, which are further optimized, is shown in Fig. 3a. These inventions solve four key problems inherent to prime editing: low efficiency at some loci, limitations due to mismatch repair, inefficient protein production, and variability in pegRNA performance. The addition of mismatch repair modulation to the prime editing systems was one of the most radical engineering innovations [31]. Mismatch repair can recognize prime-editing heteroduplex intermediates and excise the edited strand, reverting products toward the original sequence and reducing net efficiency. PE4/PE5 address this using MLH1dn, a dominant-negative MLH1 fragment that transiently inhibits mismatch repair, thereby stabilizing edited heteroduplex outcomes and improving product purity. In Fig. 3a, PE4 is depicted as a hybrid of PE2 and MLH1dn, and PE5 is a hybrid of the MLH1dn module and PE3 dual-nick approach [7,8,31,32]. Such systems are highly efficient at enhancing editing efficiency, particularly for the materials that are substituted, but this efficiency is highly counteracted by repair [33].

The other significant development is PEmax, which is a structural optimizations prime editor with improved nuclear localization, greater reverse transcriptase stability, and codon-optimized domains, which enhance expression in mammalian systems. Optimal use of linker sequences between Cas9 and reverse transcriptase was also rationalized to boost the activity [8,23,34]. CMP-PE and CMP-PEmax are engineered prime editors designed to improve expression uniformity and functional robustness across cell types [35]. The engineering of RNA components has also been equally significant. First-generation pegRNAs were easily degraded or unstable in form, particularly around the primer binding site or reverse transcription template. Later designs added stabilizing structural motifs, better secondary structures or 3 ends protective sequences. Enhanced pegRNAs (epegRNAs) can be of great significance in elevating the rate of editing by avoiding premature degradation. RNA stability is therefore proving to be among the strongest vectors that can enhance prime editing ability [[36], [37], [38], [39]].

Another highly important innovation is promoting targeting versatility by developing engineered versions of Cas9. Fig. 3b points out among the variants of PAM-flexible, which comprise VQR, VRQR, VRER, SpG, and SpRY, with progressive PAM promiscuity [10]. However, PAM relaxation increases the number of potential genomic binding sites and may elevate off-target exposure; in prime editing, this primarily raises concern about unintended nicking and by-product formation at permissive sites, making guide selection and off-target profiling especially important when using SpG/SpRY-based systems. These variants significantly increase access to genomic loci, and prime editors have access to previously inaccessible sequences [9,15,40]. Primarily with the capability of SpRY, allowing nearly PAM-agnostic genome-wide editing, with prime editing, these variants significantly expand targetability, enabling editing at many previously inaccessible genomic sites [41]. A combination of these engineering innovations indicates that prime editing is not a fixed invention, but a progressive platform that gets improved daily. With every iteration, there is enhanced precision, strength and applicability. Advanced prime editing systems expand the range of biological applications by incorporating protein engineering, RNA biology, and control of DNA repair, allowing the field to reach therapeutic-grade reliability. It is also a direction of this type of engineering that prepares the next generation of prime editing platforms that gradually move beyond incremental optimizations to radically new editing functionality [[42], [43], [44], [45]].

## 4 Next-generation prime editing platforms

**Fig. 4:**
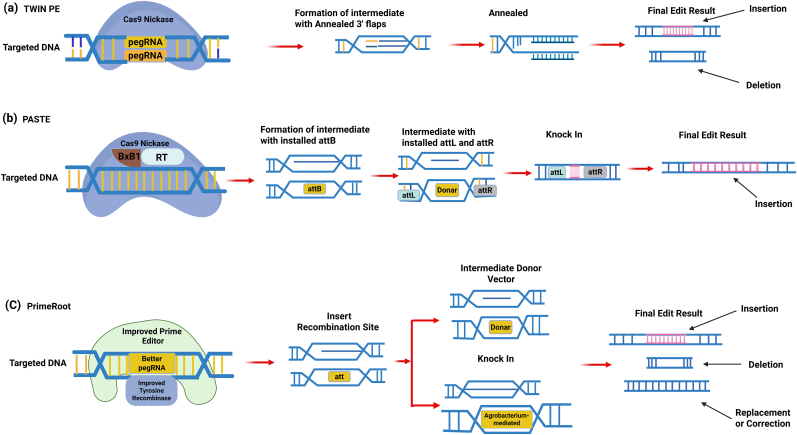
**Mechanisms of advanced prime-editing platforms. (a)** TwinPE uses paired pegRNAs to form complementary flaps, enabling precise insertions or deletions. **(b)** PASTE installs recombination sites via Bxb1-integrated prime editing for targeted sequence insertion. **(c)** PrimeRoot combines improved pegRNAs and recombinase activity to mediate insertions, deletions, or corrections.

Prime editing technologies have advanced beyond the original system and now enable large-fragment insertions, programmable recombinase integration and multi-step genome editing processes. These next-generation editors are a conceptual extension of prime editing's capabilities to the level of a programmable system of genome engineering that can support more and larger edits. Some of these expanded systems, such as TWIN-PE, PASTE, and PrimeRoot, are examples [11,44] as illustrator Fig. 4.

TWIN-PE proposes a dual pegRNA system in which synchronized flap intermediates are utilised to create big deletions or inserts. TWIN-PE allows the generation of intermediate DNA structures annealing to target predefined structures by designing pegRNAs that form coordinated 3′flaps, which result in the generation of larger edits compared to traditional prime editing strategies. TWIN-PE is best used in gene-tagging experiments, regulatory element restructuring and intermediate-scale genomic remodeling between small-base editing and full integrase-mediated insertion [11,43,46,47] as shown in Fig. 4a. PASTE is a significant concept advance, which involves prime editing on one hand and site-specific recombinases on the other. In PASTE, the prime editor integrates the bases of the reverse-transcribed bases as sites of short attachment (attB or attP) into the genome [45,46]. After the landing site is installed, a separate donor payload carrying the matching attachment site can be delivered (e.g., as a donor vector), enabling site-specific integrase-mediated insertion of kilobase-scale DNA. Such sites serve as landing sites for serine integrases that can insert large DNA fragments, such as complete genes or therapeutic cassettes, once installed. This process can be edited in blocks of kilobases to enable the smooth incorporation of high specificity payloads and eliminate any double-stranded breaks. PASTE thereby converts the prime editing to a modular integration platform that is capable of targeted gene addition [11,12,48] as shown in Fig. 4b. PrimeRoot also extends those functions further by adding a better prime editor to tyrosine recombinases. It forms recombination active sites, which allow later integration of donor vectors Thus, PrimeRoot is a two-step strategy, prime editing establishes the recombination context, and subsequent donor vector delivery enables targeted insertion or replacement. PrimeRoot models prime editing as an initial step in a multi-layered cascade of genome editing, whereby the initial edit preconditions more complex genetic edits. These platforms function as stepwise genome-rewriting systems, in which one edit can initiate subsequent modifications [21,49,50] as shown in Fig. 4c. Because these platforms are stepwise, the final successful payload-insertion rate reflects the combined efficiency of landing-site installation and downstream integrase/recombinase insertion and can therefore be lower than single-step prime-editing outcomes (Table 1). These technologically sophisticated systems combine to transform simple installations of edits to the programmable architecture of genomes, redesigned [23]. These approaches position prime editing as a foundational tool for multi-step genome engineering. An iteratively assembled diversity of RNA-guided targeting, template-guided rewriting of DNA, along with recombinase-guided integration, form future generations of prime editors amenable to modification to increasingly sophisticated genome engineering processes across therapeutic, agricultural, and synthetic biology applications [51,52].

**Table 1:** Overview of the representative CRISPR and Prime Editing Systems.

Category	System/Variant	Source or Engineering	Key Molecular Features/Modifications	Editing Mechanism & Repair Dependency	Editing Capacity	Reported Efficiency/Output	Applications	Limitations	Ref
**CRISPR/Cas9 Genome Editing**	Wild-type Cas9	*Streptococcus pyogenes*	Two nuclease domains (HNH + RuvC)	Creates DSBs via NHEJ/HDR	Knockouts, knock-ins	Moderate; cell-type dependent	Functional genomics, mutagenesis	Off-target DSBs, mosaicism	[[1], [2], [3]]
**Cas9 Nickase (H840A/D10A)**	Point-mutated Cas9	Single nuclease domain	Generates a single-strand nick	HDR or repair synthesis	Gene correction; low indels	1-10 × safer than Cas9	Precise editing	Lower yield than DSB-based CRISPR	[4]
**Cytosine Base Editor (CBE)**	Cas9 nickase + APOBEC + UGI	Engineered fusion	Converts C→T via deamination	BER/MMR dependent	Transition edits (C→T)	15-60%	Plant & animal SNP correction	Transition-only scope	[5]
**Adenine Base Editor (ABE)**	Cas9 nickase-TadA	Directed evolution	Converts A→G	DNA repair guided	Transition edits (A→G)	20-70%	Monogenic disease models	GC-locus bias	[6]
**Prime Editor (PE1-PE3b)**	Cas9(H840A)-RT + pegRNA	RT fusion, pegRNA/primer design	Reverse transcription of the edit flap	Nicked-strand synthesis, flap competition	All 12 base substitutions + small indels	Up to ∼50%, locus-dependent	Therapeutic & plant edits	pegRNA instability, context-dependent	[7]
**MMR-Inhibited Prime Editors (PE4-PE5)**	PE2/PE3 + MLH1dn	MMR suppression	Prevents WT reversion	Heteroduplex stabilization	Multi-base edits	2-10 × higher vs. PE2	Mammalian & stem-cell editing	Temporary genome-instability risks	[8]
**PEmax (Optimized Prime Editor)**	Codon-optimized PE2	Protein engineering	Enhanced NLS, optimized linkers	Same as PE2 (higher efficiency)	All PE edit types	Broad improvement across loci	Mammalian editing	Large size limits delivery	[8]
**PAM-Expanded Editors (SpG/SpRY)**	Engineered PAM residues	PAM mutagenesis	Recognize NGN/NNN PAMs	Same as PE/Cas9	Broad genome accessibility	Expanded targeting range	Editing rare loci	Relaxed PAM increases off-targets	[9,10]
**Twin Prime Editing (TWIN-PE)**	Dual pegRNAs	Paired-flap engineering	Large deletions, replacements, inversions	Dual RT-flap synthesis	Multi-kb programmable edits	Efficient but locus-dependent	Genomic rewiring, regulatory edits	Complex pegRNA design	[11]
**PASTE (PE + Integrase)**	Prime editor + integrase	Recombinase engineering	Installs landing pads	RT flap + integrase recognition	Large DNA inserts	Variable by cell type	Gene therapy, synthetic circuits	Mis-integration risks	[12]

## 5 Applications of prime editing across biological domains

**Fig. 5:**
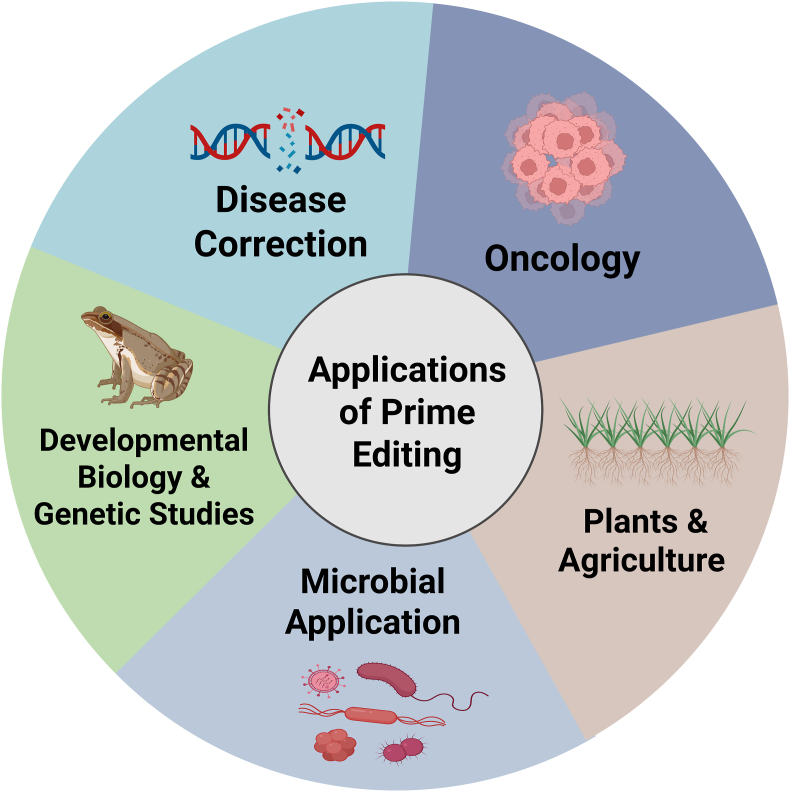
**Applications of Prime Editing.** Overview of major fields where prime editing is applicable, including disease correction, oncology, plant engineering, microbial systems, and developmental biology.

Reported efficiencies vary widely across loci, cell types, delivery formats, and chromatin contexts. As summarized in Table 1, PE2-PE3 can reach ∼50% in favorable contexts but remain locus-dependent; PE4-PE5 can improve outcomes several-fold relative to PE2; and integrase/recombinase-augmented platforms such as PASTE and PrimeRoot show substantial cell-type- and chromatin-dependent variability (Table 1). The flexibility of prime editing is broadly applicable in biological and translational research [8,42]. Key applications span disease correction, oncology, plant biotechnology, microbial engineering, and developmental biology (Fig. 5). These areas particularly benefit from the high precision and minimal genomic disruption associated with prime editing. In the context of human genetic disease, prime editing can rewrite pathogenic alleles without donor DNA templates and without creating double-strand breaks. Its capacity to install precise base substitutions, correct frameshift mutations, and repair regulatory sequences suggests that a large number of monogenic disorders could in principle be targeted. Moreover, the reduced likelihood of unintended repair outcomes relative to nuclease-based editing enhances its suitability for therapeutic use. Although delivery remains a major challenge, ongoing development of viral vectors, lipid nanoparticles, and split-prime-editor systems is gradually bringing clinical translation closer to reality [51,[53], [54], [55], [56]].

In cancer biology, prime editing can be used to accurately model cancer-associated mutations, enabling tumors genotypes to be reproduced with greater fidelity than before. Beyond modelling, prime editing can rewrite oncogenic regulatory elements, install synthetic safety switches, and engineer therapeutic immune cells. For example, prime editing offers opportunities to optimize CAR-T and other cell-based immunotherapies by introducing beneficial mutations and attenuating pathways that drive exhaustion or toxicity. Compared with nuclease-based editing, the lower risk of chromosomal rearrangements is especially advantageous in the context of immunotherapeutic engineering [56,57].

In agriculture, prime editing has broad potential because it can introduce precise genetic modifications into plants without incorporating foreign DNA, aligning with global efforts toward non-transgenic crop improvement. Traits such as stress tolerance, disease resistance, nutrient-use efficiency, and domestication of wild species can, in principle, be fine-tuned with nucleotide-level precision. Given the repetitive nature of many crop genomes and the presence of recalcitrant loci, the expanded PAM flexibility and enhanced performance of advanced prime-editing systems offer distinct advantages for plant biotechnology [50,58,59].

Microbial engineering is another promising area of prime editing. This ability to precisely modify genes, metabolic pathways, and regulatory elements can increase biomanufacturing, reverse antibiotic resistance, and introduce innovative synthetic biology [60]. Microbial genomes are typically smaller, easier to manipulate and due to being such a good model to test our ideas of advanced prime editing, such as multiplexed pegRNA targeting or multi-step cascades of genome rewriting. Precise manipulation of regulatory elements and developmental genes is made possible by prime editing in the areas of developmental biology and genetic studies. This is critical, especially when studying gene function in embryos or model organisms, as it is essential to minimize off-target effects and maintain genome integrity. The scarless character of prime editing permits a more refined assessment of the developmental phenotypes and lineage search. In all of these areas, prime editing is not only a flexible tool but also a platform whose functions can be widened with further innovation. It is considered one of the most influential genome engineering tools of this decade because it is so general across a wide range of organisms and biological problems [15,49,51,[61], [62], [63]].

## 6 Limitations and challenges

Nonetheless, despite its astute development, prime editing still faces critical limitations that can be overcome to bridge the gap to its use and therapeutic translation. The final incorporation of the comparatively large prime editor protein is also the most important limitation. Recent delivery methods, such as viral delivery, lipid nanoparticles, and mRNA-based delivery, all have challenges regarding efficiency, biodistribution, and transient expression. Future solutions could adopt split-intent approaches and smaller, designed variants. The other issue is the inconsistency in the efficiency of editing various genomic loci [[64], [65], [66], [67], [68]]. Variations in chromatin accessibility, local sequence context, pegRNA stability, and mismatch repair interactions may have a significant effect. The effective design of the best pegRNA is a complex task that requires the use of advanced computer programs and experimentation. Another source of unpredictability arises from thermodynamic competition between 3′ and 5′ flaps [7,69,70].

Although the off-target effects are usually lower compared to nuclease-based editing, they still pose a risk. These are either random reverse transcription, incorporation of incorrect nucleotides, or low-rate insertions and deletions. High fidelity is crucial to the therapeutic uses, and both protein and RNA parts have to be engineered further. The other significant challenge is the mismatch repair interactions. Although mismatch repair is inhibited to augment editing efficiency, it also has the possibility to create a threat of genome instability in case the issue of overexpression is not closely regulated [20,[71], [72], [73]]. One of the future directions is to come up with inducible and reversible or locus-specific MMR modulation strategies. Lastly, with the advanced systems, large-scale genome changes are still limited, although it is possible. The technologies help increase cargo volume, such as PASTE and PrimeRoot, but their performance varies by cell type and locus. It will be vital to develop more predictable large-fragment integration systems in applications where genomic rewrites are needed in large organizations [44,48,74].

## 7 Discussion and future perspectives

Prime editing can be positioned as a practical middle ground between nuclease-based CRISPR and base editing: it achieves template-directed rewriting without requiring double-strand breaks, reducing the risk of indels and large-scale rearrangements that can accompany break-based editing [7,[75], [76], [77]]. At the same time, it offers broader edit versatility than base editing, although performance is often more sensitive to pegRNA design, local sequence context, and the balance of cellular repair pathways [[78], [79], [80]]. Across systems and targets, editing outcomes can vary substantially by locus and cell type, and mismatch repair is a major determinant of whether edited heteroduplex intermediates are retained or corrected back toward the original sequence [81,82]. Approaches that suppress mismatch repair can increase editing yields, but they also introduce an important trade-off between efficiency and maintaining native genome surveillance, which is especially relevant when considering translational and clinical settings. Thus, prime editing's overall promise lies in combining precision with flexibility, but its most consistent deployment will depend on continued tuning of repair interactions and delivery constraints [[83], [84], [85], [86]].

As prime editing expands beyond small substitutions and indels, newer architectures such as TWIN-PE, PASTE, and PrimeRoot offer a path toward larger, more programmable genome modifications compared with conventional HDR-style large insertions, which are often inefficient and can impose substantial cellular stress. Even with these advances, integration outcomes can remain variable across cell types and loci, and are influenced by chromatin accessibility and cell-cycle state, emphasizing that “editability” is not purely sequence-defined [11,[87], [88], [89]]. In addition, large-payload strategies ultimately depend on integrase specificity, insertion fidelity, and predictable outcome control, which remain core requirements for broad adoption and clinical-grade reliability. In plant systems, prime-editing efficiencies often lag behind those in mammalian contexts, reinforcing the need for organism- and tissue-specific optimizations strategies for routine deployment in agricultural biotechnology. Together, these observations suggest that while prime editing is maturing rapidly, its performance envelope is still shaped by biological context, and systematic benchmarking remains essential as more complex platforms emerge [[90], [91], [92], [93], [94]].

Looking forward, three priorities are likely to define the next phase of progress. First, improved molecular and RNA design through refined editor architectures and pegRNA engineering should help raise efficiency and reduce unwanted by-products in a more predictable, generalizable manner [95,96]. Second, continued development of integrase-and recombinase-assisted strategies is expected to increase the feasibility of installing larger DNA segments without double-strand breaks, but these methods must achieve greater consistency across chromatin states and cell types, and must demonstrate robust specificity and product fidelity [[97], [98], [99]]. Third, delivery remains a central bottleneck because prime editors are large; therefore, scalable solutions such as split systems and improved delivery vehicles will be critical for in vivo applications and tissue-specific editing [100,101].

In parallel, AI-assisted tool development is becoming increasingly relevant: machine learning and computational protein design can support pegRNA prioritisation, outcome prediction, off-target risk reduction, and the engineering of improved Cas9 and reverse transcriptase components. However, predictive models must be trained and validated across diverse experimental conditions, because limited or biased datasets can reduce generalizability across species, cell types, and chromatin environments. Finally, ambitious goals such as genome-scale rewriting and extensive multiplexing will require major gains in efficiency and predictability, since current constraints repair competition, cumulative burden, and context-dependent heteroduplex processing, still limit reliable scaling [69,81,102,103].

In conclusion, prime editing has developed into a highly adaptable genome-engineering platform that expands precise editing beyond what is readily accessible with nuclease editing or base editing alone. Continued progress in pegRNA/editor engineering, integration strategies, and delivery technologies is expected to improve robustness across biological contexts and accelerate practical translation. With stronger predictive design supported by AI and better benchmarking, prime editing is positioned to become a central toolkit for next-generation research and applied biotechnology.

## Informed consent statement

Not applicable.

## Author contributions

Waqar Muhammad and Ali Amjad, as co-first authors, contributed equally to the conceptualization, investigation, and drafting of the original manuscript. Yufei Liu and Aziz Umar provided critical input through review, editing, data curation, and validation. Kai Chen contributed to the figure drawings. Xiaolong Wang reviewed the draft and provided many constructive suggestions. Kun Xu, as the corresponding author, supervised the project, secured funding, monitored its progress, and was responsible for the final review and editing of the manuscript. All authors have read and approved the final version of the manuscript.

## Funding

This work is supported by the Agriculture Science and Technology Major Project and the Biological Breeding-Major Projects (grant nos. 2023ZD04074 and 2023ZD04051).

## Declaration of competing interest

The authors declare that they have no known competing financial interests or personal relationships that could have influenced the work reported in this review.
